# Protective Effect of Melatonin against Inequality-Induced Da mages on Testicular Tissue and Sper m Para meters 

**Published:** 2013-12-22

**Authors:** Shiva Nasiraei-Moghadam Nasiraei-Moghadam, Kazem Parivar, Abolhasan Ahmadiani, Mansoureh Movahhedin, Mohammad Reza Vaez Mahdavi

**Affiliations:** 1 Department of Biology, Faculty of Basic Sciences, Science and Research Branch, Islamic Azad University, Tehran, Iran; 2 Neuroscience Research Center, Shahid Beheshti University of Medical Sciences, Tehran, Iran; 3 Department of Anatomy, Faculty of Medical Sciences, Tarbiat Modares University, Tehran, Iran; 4 Department of Clinical Trial Iranian Traditional Medicine Research Center and Health Equity, College of Medicine, Shahed University, Tehran, Iran

**Keywords:** Testis, Melatonin, Food deprivation, Isolation situation

## Abstract

**Background:**

The goals of the study are evaluation the effects of food deprivation and isolation situation as a social stress on fertility; and in the following, investigation of the improving effect of melatonin as an antioxidant component.

**Materials and Methods:**

In this experimental study, We investigated histopathological and serological effects of melatonin and social stress (food deprivation and isolation) on different features of sperm and testicular tissue among 42 male rats in 7 groups including control, sham, melatonin received (M), food deprivation (FD), Food deprivation and melatonin treatment (FDM), Food deprivation and isolation situation (FDi), and Food deprivation and melatonin treatment and isolation situation (FDMi) groups. Epididymal sperms of all rats were also counted. Histopathological evaluation of the testes was done under a light microscopy to determine the number of spermiogenic cells. Serological evaluation of testosterone, corticosterone, and melatonin was performed, as well. For statistical analysis, oneway ANOVA and Tukey’s post hoc test were used, and the value of p≤0.05 was considered statistically significance.

**Results:**

The result showed that food deprivation increased the number of abnormal, immotile, and dead sperms, while decreased the number of normal sperms (p<0.05). Isolation could improve sperm motility and viability, while enhanced the number of sper- matogenic cells. Melatonin had a protective effect on sperm count, motility, and viability, while reduced sperm abnormality.

**Conclusion:**

Our results demonstrated that melatonin treatment and isolation situation improve the parameters related to epididymal sperms and spermatogenic cells after food deprivation.

## Introduction

Food deprivation, as a cycle, is strongly related to different aspects of social health (1). In many societies, poverty is related to high death rate and low life hope (2-4). However, the precise relationship between socioeconomic status and many aspects of health such as fertility remains unclear.

The infertility in adult males is estimated to be six percent (5). Anatomical abnormalities including ductal obstructions, varicocele, or ejaculatory disorders contribute in the infertility of adult males, however, a big portion (40-90%) of cases are believed to be caused by deficient sperm production (6). Sperm production is the main function of male fertility, and many different factors can affect this feature (7-9). Therapeutic and non-therapeutic agents accompanied with environmental factors, such as chemicals and radiation, are able to regulate or affect sperm production (10).

Many studies have found stress as one of the factors that affects fertility. As compagne suggested, stress may influence fertility through some neurobiological pathways (11). In general, stress and its outcomes are associated with hypothalamic-pituitary-adrenal (HPA) axis.; in addition, the effect of stress on fertility is related to gonadal function, so these two endocrine systems operate interactively. This relationship in male rats reveals that testosterone can influence different aspects of basal and specific HPA function. This interaction occurred with induction of adrenocorticotropic hormone (ACTH) release through the effect on several testosterone-sensitive afferents on the HPA axis, which leads to integrated reproductive and social behavior (12). Moreover, some studies on psychological distress have shown relationship between socio-psychological aspects and infertility. These effects have been observed in central nervous system rather than peripheral that mediated by the behavioral effects (13). However, some studies have shown that low stress level leads to better female and male natural fertility (11).

A recent study has suggested that social stress causes oxidative stress and increases production of oxidative metabolites (14). Many studies have shown that the anti-oxidative effect of pineal gland hormone, melatonin, which reacts with many oxidant reagents such as single oxygen, ozone, carbonate radicals, and reactive nitrogen species (15- 18). Also, it has the capability of scavenging free radicals at high rates in the absence of light (16, 17).

In this study, we investigated the effect of food deprivation and social status, as well as the probable therapeutic effect of melatonin on fertility health of rats. Our data reveals that melatonin could improve some adverse effect of food deprivation in male rat sperm features and testis structure. The common semen features including sperm concentration, motility, viability, and morphology are used as the standard World Health Organization (WHO) criteria in animal studies (9).

## Materials and Methods

### Animals

For this experimental study, the protocol was approved by the Research and Ethics Committee of Science and Research Deputy of Azad University, Tehran, Iran. Forty-two rats were kept under standard laboratory conditions with a 12-hour light/ dark cycle and food and water ad libitum throughout the experiments. In this study, the effects of ad libitum access to food and water and melatonin treatment were evaluated with and without food deprivation and isolation situation.

### Drug and chemicals

Melatonin powder (Sigma-Aldrich, USA) was resolved in saline and alcohol 95-5% (V/V). Xylene, ethanol, eosin, nigrosin, Papanicolaou staining and Bouin’s solutions were all purchased from Merck, Germany. Other materials used are as follows: Hams F10 + Fetal Bovine Serum (FBS, Gibco, UK), testosterone (Diagnostics Biochem Canada Inc., Ontario, Canada), corticosterone (DRG, Marburg, Germany), and melatonin enzyme-linked immunosorbent assay (ELISA) kit (Cusabio Biotech, Wuhan, China). Micro plates containing diluted serum and different concentrations of specific hormone was used to record observed optical density (OD) using Elisa reader.

### Groups

Animals in the control group (C) remained intact and were kept in the animal room during the study. They received normal food without any limitation, approximately 22 g a day. The sham control group (S) received saline as melatonin vehicle. The third group, Melatonin treatment (M), received daily intraperitoneal melatonin (5 mg/kg body weight). The remaining four groups including food deprivation (FD) under inequality condition (Inequality: FDi-FD=animals inequality. Different results between FD and FDi groups revile inequality sense), food deprivation with isolation (FDi), food deprivation with melatonin injection (FDM), and food deprivation with melatonin injection and isolation (FDMi) underwent food deprivation condition and received one-third of the normal daily food, 7.5 g/day. Isolation was the condition under which animals (6 animals in 1 cage) could not see or smell the food of other animals. The rats were weighted twice, in the beginning and at the end of the experiment.

After two weeks, blood samples were obtained from the orbital vein of the rats. Serum was separated from the blood samples by centrifugation at 5000 rpm for 5 minutes. Animals were anesthetized with CO_2_ and sacrificed, and the epididymis was removed for sperm feature evaluation. After removal and washing of the testes, they were weighted and their dimensions were measured by a caliper.

### Sperm feature evaluation procedure

Sperms were obtained from the two heads of epididymis of each rat. They were placed in 1 ml of the medium containing Hams F10 + FBS in 9/1, and incubated at 37˚C for 15 minutes. The sperm count and motility were evaluated by hemocytometer using Neubauer slide (19) and a light microscopy (Labo America Inc.,USA). Sperm viability was assessed using eosin-nigrosin staining (20, 21). Evaluation of abnormality of the sperm heads was done by Papanicolaou staining (22). Sperm tails were not evaluated because of their different characteristics and the potential risk of errors (22-24). From each rat, a total of 400 sperms were examined for morphological abnormalities.

### Histological procedure

The testis tissues were fixed in Bouin’s solution for 20 hours and prepared for histopathological evaluation. After processing and embedding in paraffin, the tissues were sectioned at thickness of 5 μm by rotary microtome (Leitz, Germany) and stained with hematoxylin and eosin, according to the standard staining protocol. Histopathological evaluation of the tissues was performed by a light microscopy (Labo America Inc., USA).

An ocular grid (with a graticule area of about 48×48 mm) was used to measure the diameters of testis sections. Also, for each testis, the numbers of spermatogenic and Sertoli-Leydig cells in 100 randomly-selected round or nearly-round tubular profiles were determined under a light microscopy. An estimate of each parameter was achieved by examining 20 fields in five histological sections from each testis (25).

Since staging was not possible in all tubules, additional patterns of number and diameter of both seminiferous tubules and tunica albuginea were determined on magnified digital pictures (26).

### Serological procedure

Peripheral blood was extracted from the orbital vein, and the serum was then separated. The serum was aliquated and kept at -70˚C. The serum samples in all groups were evaluated by testosterone (Diagnostics Biochem Canada Inc., Ontario, Canada), corticosterone (DRG, Marburg, Germany), and melatonin ELISA kits (Cusabio Biotech, Wuhan, China) using Elisa reader to record OD.

### Data analysis and comparing method

Data analysis was carried out by Statistical Package for the Social Sciences, version 16 (SPSS Inc., IL, USA). All data are presented as the mean ± SD. "Body and testis weight" were compered between groups using Mann-Whitney test. Other comparisons were done with ANOVA and Tukey’s post hoc, and the value of p<0.05 was considered statistically significant. Comparison of the control group with other groups is shown by p value1. Moreover, comparison between the FD group and the FDi, FDM, and FDMi groups is demonstrated by p value^2^.

## Results

### Body and testis weights

All rats remained in relatively good health status during the experiment period. Data on body and testis weights are presented in table 1. The percentage increase of body weight in the control group was 66%, while the percentage decrease of body weight in FD, FDi, FDM, FDMi, and M-treated animals were 26, 7, 8, 1, and 11%, respectively. There were no treatment-related changes in the absolute and relative weights of the testis in the treated groups, as compared with the control group. Since there was no statistically significant difference between the control and the sham groups, the results of the sham group were ignored.

**Table 1:** Initial and final body-weight (g) and relative organ weight (g) of rats after14 days


	Control	M	FD	FDi		FDM		FDMi	
	mean ± SD	mean ± SD	mean ± SD	mean ± SD	p-value^2^	mean ± SD	p-value^2^	mean ± SD	p-value^2^

**Initial body-weight**	192 ± 18	190 ± 20	195 ± 15	190 ± 17		190 ± 20		194 ± 15	
**p-value^1^**									
**Final body-weight**	270.16 ± 35.17	248.67 ± 9.24	190 ± 13.07	176.39 ± 6.89		178.83 ± 27.77		205.38 ± 13.48	p<0.05
**p-value^1^**			p<0.001	p<0.001		p<0.001		p<0.001	
**Testis weight**	1.46 ± 0.23	1.41 ± 0.31	1.27 ± 0.22	1.26 ± 0.17		1.35 ± 0.28		1.40 ± 0.29	
**p-value^1^**									


The data are expressed as mean ± SD (Standard Deviation) for six rats in each group. Significant difference (p<0.05) between the control group and other groups is shown by p-value1, while significant difference (p<0.05) between the FD group and the FDi, FDM, and FDM groups is shown by p value^2^.

### Sperm analysis

Table 2 provides the data on sperm motility, abnormality, dead-live ratio, and total sperm abnormality. When compared with the control animals, the percentage increase of immotile sperm in FD, FDi, FDM, and FDMi groups were 300, 45, 50, and 30%, respectively, whereas the percentage decrease of immotile sperm in the melatonin-treated animals (M) was 40%. While a significant increase in the number of dead sperm was observed in the FD group, the percentage decrease of immotile sperm in other groups was significantly compared to the FD group. The number of dead sperm significantly increased in the FD and FDi groups, while in the FDi and FDMi groups, this number significantly decreased as compared with the FD group. The number of sperms significantly decreased in the FD and FDi groups. Moreover, in the FDM and FDMi groups, a significant increase was observed in the number of sperms compared with the FD group. The percentage increase of morphologically abnormal sperms was statistically significant in the FD group. Also, in the FDM and FDMi groups, the percentage of morphologically abnormal sperm significantly decreased compared with the FD group. Table 3 shows the percentage decrease of epididymal sperm number (ESN) in the FD, FDi, FDM and FDMi groups as follows: 45, 35, 15, and 7%, respectively. Furthermore, in the melatonin-treated animals (M), the number of sperm increased by 8%.

**Table 2:** Motility, abnormal head of sperms and viability rates (%) of sperms aspirated from head of epididymis after treatment for 14 days


	Control	M	FD	FDi		FDM		FDMi	
	mean ± SD	mean ± SD	mean ± SD	mean ± SD	p-value^2^	mean ± SD	p-value^2^	mean ± SD	p-value^2^

**Sperm motility(Motile sperm) **	79.21 ± 3.27	89 ± 3.789.24	19.44 ± 3.74	77.84 ± 4.97	p<0.01	65.88 ± 5.62	p<0.01	73.5 ± 2.64	p<0.001
**p-value1**			P<0.001						
**Abnormal head of sperm**	1.83 ± 0.54	1. ± 0.60	4.08 ± 0.67	3.2 ± 0.58	p<0.05	2.18 ± 0.59	p<0.01	2.00 ± 0.46	p<0.01
**p-value1**			p<0.01	p<0.05					
**Viability(Dead sperm)**	9.17 ± 2.08	7.3 ± 1.84	88.16 ± 2.62	33.13 ± 3.84	p<0.05	25.66 ± 3.39	p<0.01	11 ± 3.05	p<0.001
**p-value1**			p<0.001	p<0.05		p<0.05			


The data are expressed as mean ± SD (Standard Deviation) for six rats in each group. Significant difference (p<0.05) between the control group and other groups is shown by p value1, and significant difference (p<0.05) between the FD group and the FDi, FDM, and FDM groups is shown by p value^2^.

**Table 3:** Sperm number per ml in head of epididymis after treatment for 14 days


Sperm count per ml in head of epididymis (mean ± SD) ×106									
	Control	M	FD	FDi		FDM		FDMi	
	mean ± SD	mean ± SD	mean ± SD	mean ± SD	p-value^2^	mean ± SD	p-value^2^	mean ± SD	p-value^2^

**Epididymal sperm Number×106 **	37.83 ± 3.26	40.66 ± 3.77	19.16 ± 3.74	25.5 ± 5.63		32.25 ± 4.96	p<0.05	35.16 ± 3.74	p<0.01
**p-value1**			p<0.01	p<0.05					


The data are expressed as mean ± SD (Standard Deviation) for six rats in each group. Significant difference (p<0.05) between the control group and other groups is shown by p-value1, while significant difference (p<0.05) between the FD group on the one hand and the FDi, FDM, and FDM groups on the other hand is shown by p value^2^.

### Analysis of morphological changes

As shown in table 4, there was a statistically significant difference between the control group and the FD group in the number of all spermatogenic cells. Moreover, the FDi, FDM, and FDMi groups showed improvement in this respect. However, melatonin treatment could not improve the number of these cells in comparison with the control group.

### Plasma hormone analysis

As the data in table 5 shows, corticosterone levels were significantly lower in the FDM and M groups than in the control group. Moreover, the testosterone levels in all groups except the M group significantly decreased. In contrast, the melatonin plasma levels in all groups significantly elevated in comparison with the control group, except for the FD group.

**Table 4:** Cell number per graticule area of 48 × 48 mm (in the control, FD, FDi, FDM, FDMi, and M groups) after 14 days


	Control	M	FD	FDi		FDM		FDMi	
	mean ± SD	mean ± SD	mean ± SD	mean ± SD	p-value^2^	mean ± SD	Ppvalue^2^	mean ± SD	p-value^2^

**Spermatogonia**	34.22 ± 10.43	38.55 ± 10.56	18.1 ± 8.43	24.62 ± 9.34	p<0.05	24.39 ± 9.565	p<0.05	27.37 ± 10.75	p<0.01
**p-value1**			p<0.001	p<0.01		p<0.01		P<0.05	
**Spermatocyte**	68.16 ± 13.4	69.44 ± 15.65	36.63 ± 12.43	49.6 ± 13.67	p<0.01	53.51 ± 12.56	p<0.01	63 ± 11.67	p<0.001
**p-value1**			p<0.001	p<0.05		p<0.05			
**Spermatid**	42.88 ± 12.43	45.83 ± 12.67	23 ± 9.56	26.51 ± 9.65		29.84 ± 10.67	p<0.01	41.61 ± 10.75	p<0.001
**p-value1**			p<0.001	p<0.001		p<0.01			
**Sertoli**	2.36 ± 0.25	2.49 ± 0.23	0.67 ± 0.225	1.04 ± 0.224		1.83 ± 0.25	p<0.001	1.85 ± 0.26	p<0.01
**p-value1**			p<0.01	p<0.01		p<0.05		P<0.05	
**Leydig**	2.01 ± 0.21	1.62 ± 0.24	0.63 ± 0.12	0.80 ± 0.18		0.77 ± 0.1		0.79 ± 0.12	---------
**p-value1**			p<0.001	p<0.01		p<0.01		P<0.01	


The data are expressed as mean ± SD (Standard Deviation) for six rats in each group. Significant difference (p<0.05) between the control group and other groups is shown by p-value1, while significant difference (p<0.05) between the FD group and the FDi, FDM, and FDM groups is shown by p value^2^.

**Table 5:** Plasma levels of hormones after 14 days


Ng/ml	Control	M	FD	FDi		FDM		FDMi	
	mean ± SD	mean ± SD	mean ± SD	mean ± SD	p-value^2^	mean ± SD	p-value^2^	mean ± SD	p-value^2^

**Corticosterone**	603 ± 125	285 ± 62	447 ± 118	445 ± 121		371 ± 90		388 ± 87	
**p-value1**		p<0.01				p<0.05			
**Testosterone**	6.18 ± 1.6	3.01 ± 1.45	1.18 ± 0.81	1.12 ± 0.56		0.55 ± 0.12		0.65 ± 0.31	
**p-value1**			p<0.01	p<0.01		p<0.01		p<0.01	

** Pg/ml**	**Control**	**M**	**FD**	**FDi**		**FDM**		**FDMi**	
	**mean ± SD**	**mean ± SD**	**mean ± SD**	**mean ± SD**	**p-value^2^**	**mean ± SD**	**p-value^2^**	**mean ± SD**	**p-value^2^**

**Melatonin**	39.3 ± 10.6	63 ±7.9	50.6 ± 8.89	59.2 ± 11.87		60.6 ± 11.87		65 ± 0.31	
**p-value1**		p<0.01		p<0.05		p<0.05		p<0.01	


The data are expressed as mean ± SD (Standard Deviation) for six rats in each group. Significant difference (p<0.05) between the control group and other groups is shown by p value1, while significant difference (p<0.05) between the FD group and the FDi, FDM, and FDM groups is shown by p value^2^.

## Discussion

Food deprivation as well as the perception of inequality in food as a social stressor can cause cell damage. In this research, we inducted food deprivation in two different situations; first, the inequality-sensed group which were housed in the same room which controls and other animals were living, so that they could sense (see and smell) other rats feeding, and second, food deprivation under isolated condition in which the isolated animals (six in one cage) were housed in an isolated room and could not smell and see other groups (1). In this study, under isolation condition, the animals suffered only from limitation in food intake, whereas the latter suffered from both "food intake limitation" and "inequality". It can be stated that when animals compare themselves with other groups and watch other feeding, it puts them in an additional stress condition. Previously, we have shown that inequality could worsen the function of different tissues in different species compared to isolated food deprived and control animals (1-4).

Our findings showed that food deprivation as a stressor decreased the corticosterone levels in comparison with those under normal conditions, conflicting to expectation that stress usually elevates corticosterone levels (27, 28). This may be due to corticosterone-dependent effect on the body condition (29, 30), whereas increased glucocorticoid production in response to chronic stressors requires more energy than in response to acute condition (31). Furthermore, stressful conditions when enough food is available increase concentration of corticosterone. Cote et al. (32) found that a variety of behavioral and physiological responses are induced by corticosterone, depending on the availability of food. In other words, catabolic state in food deprivation modifies the general response of the organism to stress in this situation and prevents increasing of corticosterone seeking energy reservation. (4, 27-29, 32, 33). Moradi et al. (4) have shown social instability increases serum corticosteroid levels, whereas food deprivation not only fails to show this effect, but decreases the serum corticosteroid. Consistently, we found that there was small decrease in the plasma concentration of corticosterone in both food deprived groups compared with the controls.

Melatonin is a potent antioxidant agent and a direct scavenger of toxic hydroxyl radical. Also, it stimulates the activity of glutathione peroxidase, which is an antioxidant enzyme (34-36). Therefore, we used melatonin as an antioxidant component for treatment of the experimental groups (stress condition caused by food deprivation). Our findings showed that melatonin has a protective effect on sperm number, motility, and viability, while can reduce sperm abnormality. Recently, it has been reported that melatonin administration can prevent the increase in plasma homocysteine (Hcy). Hcy causes oxidative damage (28) and inhibits the activity of plasma antioxidant enzymes (37, 38). Administration of melatonin can prevent adverse effects of Hcy (37, 38). Also, it has been shown that melatonin could stimulate testis growth and positively improves testicular injury (39).

**Fig 1:**
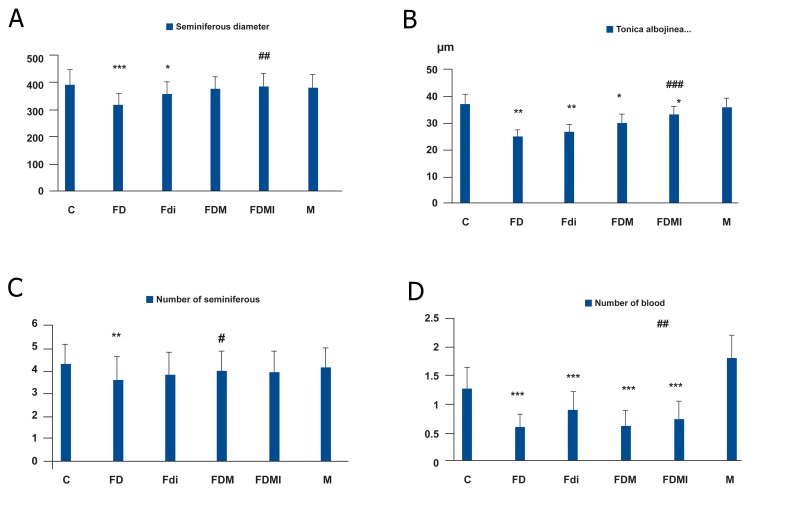
The seminferous diameter (A) and Tonica albojinea thickness (μm, B) by magnified digital picture, and the number of seminiferous tubules (C) and number of blood vessels by graticule area (about 48 × 48 mm2) in testis tissue sections. The data are expressed as mean ± SD (Standard Deviation) for six rats in each group. Significant difference (p<0.05) between the control group and other groups is shown by (*; p<0.01, **; p<0.001 and ***; p<0.05),, whereas significant difference between the FD group and the FDi, FDM, and FDM groups is shown by (# ; p<0.01, ##; p<0.001 and ### ; p<0.05).

**Fig 2:**
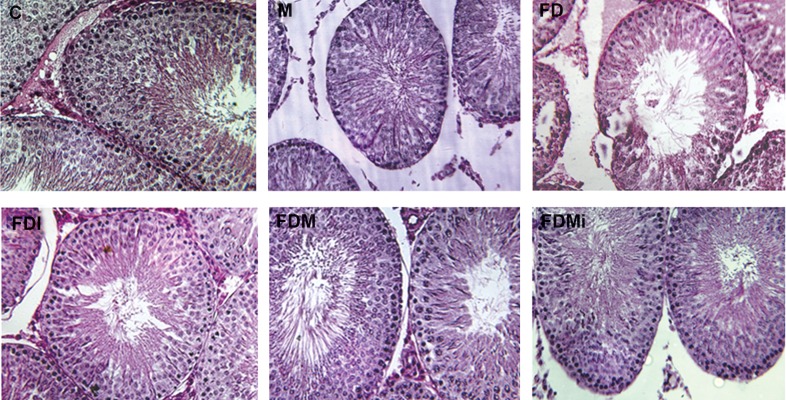
Testis cross section: control group (C), melatonin group (M), food deprvation (FD), food deprivation with melatonin (FDM), food deprivation with isolation (Fdi) and food deprivation with melatonin and isolation(FDMi), by H&E staining and ×400 magnification. FD figure shows the center of seminiferous tubules are almost empty of sperm, while FDi, FDM and FDMi figures show the seminiferous tubules center contain sperm, similar to control and M groups.

Spermatogenesis is affected by some environmental factors such as radiation and chemicals, which can regulate or affect sperm production (10). In this study, it was shown that food deprivation causes intensive reduction in spermatogenic cells, while melatonin could improve the number of these cells. In addition, food deprivation reduced the number of Sertoli-Leydig cells as non-spermatogenic cells, while melatonin treatment could prevent only reduction in the number of Sertoli cells. In contrast, blood vessels, tunica albuginea thickness, and the number of seminiferous tubules were not affected by melatonin treatment. It seems that melatonin is more effective on mitotic and spermiogenic cells and has little effect on non-spermiogenic cells such as Leydig cells or testis structures (Figs 1, 2). Some studies have shown that melatonin is a hydroxyl radical scavenger that protects DNA from free radical attacks (40). So, it is expected that melatonin demonstrates higher protective effect on mitotic cells as we observed.

In addition, we observed that the plasma level of testosterone decreased after exposure to the stressor, which is in agreement with the findings reported in other studies (41). This may be due to the decrease in the number of Leydig cells, which was not improved by melatonin administration. However, we observed a light decrease in the plasma testosterone concentration, even in the melatonin-treated rats (M group). Also, a human study demonstrated a decrease in plasma testosterone level by melatonin treatment at middle ages (42). However, in adult rats (70-90 days old), melatonin treatment caused only a slight decrease in the plasma testosterone concentration (43).

In this study, we also investigated the improvement effect(s) of isolation in the experimental groups. Here, we observed differences between the food-deprived group and isolated group experiencing food deprivation. Our results showed that isolation situation improves sperm motility and viability, and also increases the number of spermiogenic cells (spermatogonia and spermatocytes). It has also a positive effect on the plasma level of melatonin. These observations indicate that isolation is useful to elevate the level of melatonin hormone as an antioxidant component (37, 38).

As in previous studies reported that social skills and psychological factors related to depression could affect infertility or acceptance-rejection fertility in human couples (44, 45); our study on experimental animals also confirmed the same psychological effects.

## Conclusion

Our findings demonstrated that food deprivation with inequality increased the number of pathologic, immotile, and dead sperms, while decreased the total number of sperms. In contrast, isolation situation means food deprivation without inequality improved sperm motility and viability and enhanced the number of spermatogenic cells compared to food deprived group.

According to the differences observed between the FDi and FD groups in spermatogenic cells and sperm features (i.e., motility, viability, count, and morphology), it is suggested that inequality is responsible for the negative effects of food deprivation.

The results of this study also showed that melatonin treatment together with food deprivation had a protective effect on sperm number, motility, and viability. It also reduced the number of sperms with abnormal morphology and improved parameters related to epididymal sperms and spermatogenic cells. Mean while, the results showed no significant change in the number of seminiferous tubules or blood vessels and tunica albuginea thickness under isolated situation.

The next step would be investigating the pathways through which these factors affect epididymal sperms and the structure of testis.
